# Evaluation of a 12-week lifestyle education intervention with or without partial meal replacement in Thai adults with obesity and metabolic syndrome: a randomised trial

**DOI:** 10.1038/s41387-018-0034-0

**Published:** 2018-04-25

**Authors:** Kusuma Chaiyasoot, Rungnapha Sarasak, Banchamaphon Pheungruang, Suwitcha Dawilai, Pornpoj Pramyothin, Adhiratha Boonyasiri, Orawan Supapueng, Friedrich C. Jassil, Preyanuj Yamwong, Rachel L. Batterham

**Affiliations:** 1 Division of Clinical Nutrition, Department of Medicine, Faculty of Medicine Siriraj Hospital, Mahidol University, Bangkok, Thailand; 2 Department of Medicine, Centre for Obesity Research, University College London, London, UK; 3 Research Centre of Nutrition Support, Faculty of Medicine Siriraj Hospital, Mahidol University, Bangkok, Thailand; 4 Division of Clinical Epidemiology, Department of Research and Development, Faculty of Medicine Siriraj Hospital, Mahidol University, Bangkok, Thailand; 5 National Institute of Health Research, University College London Hospitals Biomedical Research Centre, London, UK

## Abstract

**Background/Objectives:**

There have been no studies examining the efficacy of meal replacement (MR) on weight loss and metabolic syndrome (MS) improvement in Southeast Asians. Thus, we undertook a 12-week randomised trial to evaluate the effect of a lifestyle education intervention alone (LEI) or with partial MR (LEI + MR) in obese Thai adults with MS.

**Subjects/Methods:**

A total of 110 patients were randomised to receive either LEI or LEI + MR. Both groups received LEI to achieve weight loss. LEI + MR group additionally received two MR daily to replace either breakfast, lunch or dinner. Mean ± SE body mass index of all participants was 34.6 ± 0.6 kg/m^2^, mean ± SE age was 42.5 ± 1.1 years and 83% of patients were female. Both groups were compared for anthropometric and cardiometabolic indices at 12-week. Body weight was also compared at weeks 38 and 64.

**Results:**

At 12 weeks, both groups exhibited statistically significant percentage weight loss (%WL) compared to initial weight but greater %WL was observed in LEI + MR compared to LEI, 2.9% vs. 1.5%, respectively (*p* < 0.05). MS criteria such as waist circumference and blood pressure improved significantly in both groups compared to baseline. However, improvement in fasting plasma glucose (FPG) was only significant in LEI + MR, and more participants with impaired FPG at baseline in LEI + MR (42.9%) than LEI (19%) returned to normal FPG at 12 weeks (*p* < 0.05). HbA_1c_, fasting insulin and HOMA-IR in LEI + MR were significantly lower than with LEI. At the end of the 12-week intervention period, 16% of participants no longer fulfilled MS criteria. A statistically significant weight loss from baseline persisted until 38 weeks but no longer reached statistically significant difference between groups

**Conclusions:**

LEI and LEI + MR were acceptable and led to improvement in weight and MS. LEI + MR group exhibited additional weight reduction and glycemic benefits at 12 weeks.

## Introduction

Obesity is a chronic relapsing medical condition that increases the risk of developing several diseases including type 2 diabetes (T2DM), cardiovascular disease (CVD), certain types of cancer, psychological problems and reduces life expectancy^1^. Metabolic syndrome (MS), a condition commonly found in obesity^2^ and proposed to be driven primarily by insulin resistance, is a constellation of metabolic derangements including abdominal obesity, elevation of blood pressure (BP), fasting plasma glucose (FPG) level, triglyceride (TG) level and low level of high-density lipoprotein cholesterol (HDL-c). It is also significantly related to the risk of the development of T2DM and CVD^3^.

Globally, the prevalence of obesity continues to increase with over 600 million (13%) adults affected by obesity in 2014^4^. In particular, the prevalence of obesity and MS have markedly increased in many low-income and middle-income countries^5^, as a consequence of economic growth, increased urbanisation and adoption of a ‘western’ lifestyle^6^. One such country is Thailand, where there has been a marked increase in obesity, defined as a body mass index (BMI) of 25 kg/m^2^ or more^7^, and MS with 37.5% and 28.9% of the adult population effected, respectively^8^.

Weight-loss interventions aimed at reducing weight at least 3–5%^9^ by reducing energy intake and increasing physical activity through behavioural modification are the mainstay of the treatment of obesity and MS^10^. However, poor compliance is the key obstacle^11,12^. Currently, there is a paucity of data regarding the effectiveness of weight-loss programmes in Thai adults coupled with a lack of specialist weight management services, hampering the delivery of multi-disciplinary intensive lifestyle interventions akin to the Look AHEAD study^13^. A previous study by Karintrakul et al.^14^ demonstrated that four monthly individualised nutrition counselling visits with 3, 5–10-min telephone contacts led to a significant weight reduction in Thai women with overweight and obesity.

Meal replacement (MR), either partial or full have been proposed to be an effective strategy assisting patients to lose weight and gain metabolic advantages in western countries^13,15–22^ and some subgroups of Asian ethnicities; Indians^23^, Chinese^24^, Japanese^25^ and Koreans^26^. However, there are no data examining their effectiveness in Southeast Asians. Given the high prevalence of obesity and MS within Thailand, there is an urgent need to develop and evaluate pragmatic cost-effective weight management programmes that can be delivered at scale. Incorporating partial or full MR into a weight management programme incurs additional economic costs. Thus, we undertook a randomised trial in Thai adults with obesity and MS to evaluate the effect of a low-cost lifestyle education intervention (LEI) alone or a LEI with MR (LEI + MR) on body weight (BW), body composition, insulin sensitivity and metabolic variables.

## Subjects and methods

### Study design and setting

The Siriraj Institutional Review Board, Mahidol University, Bangkok, Thailand reviewed and approved the trial protocol for this single-centre prospective randomised trial. The trial has been registered in the clinical trial registry of the National Institute of Health (Reference: NCT02626741). Potential subjects were alerted to the trial through the placement of posters on notice boards throughout the Siriraj Hospital and were advised to contact the study team by telephone for further information and initial screening. Interested individuals fulfilling initial inclusion and exclusion criteria then attended a visit at the Research Centre of Nutrition Support, Faculty of Medicine Siriraj Hospital, Mahidol University, between February 2015 and December 2015 and were given a participant information sheet and verbal explanation from the study investigators in person. Participants subsequently provided written informed consent prior to undergoing a medical examination and baseline blood chemistry analysis.

The inclusion criteria were adults aged ≥18 years old with BMI ≥25 kg/m^2^, fulfilling the International Diabetes Federation definition for MS^27^, which includes waist circumference (WC) of ≥90 cm if males and ≥80 cm if females plus any two of the following four factors:

1. Raised TG level of ≥1.7 mmol/L or receiving specific treatment.
2. Reduced HDL-c level of <1.03 mmol/L in males and <1.29 mmol/L in females or receiving specific treatment.
3. Raised BP, systolic BP ≥130 or diastolic BP ≥85 mmHg or receiving treatment for hypertension.
4. Raised FPG level of ≥5.6 mmol/L or previously diagnosed T2DM.

Subjects were excluded if their medications were adjusted over preceding 3 months or had uncontrolled T2DM defined as HbA_1c_ > 53 mmol/mol, short bowel syndrome, active CVD, renal impairment, abnormal liver function test and full blood count, alcohol dependence, drug abuse, pregnancy, lactation, food allergy and had metallic implants or pacemakers which is contraindicated for bioelectrical impedance analysis (BIA).

Following baseline assessments, participants who met the eligibility criteria were randomly assigned (1:1 allocation) to receive either LEI or LEI + MR by a computer-generated block randomisation. Opaque concealed envelopes were drawn by independent personnel who was not involved in the study to ensure allocation concealment. Neither the investigators nor the participants were blinded to the group allocation due to the nature of the intervention.

### Interventions

Both groups received lifestyle education delivered by a dietitian, which comprised a group session at baseline followed by four individual sessions at weeks 2, 4, 8 and 12. The main objective of the lifestyle education was to raise participants’ awareness regarding the health complications related to obesity and MS and subsequently to promote weight loss through nutritional and behavioural modifications.

The group educational session, attended by 15–20 participants, focused upon ways to reduce energy intake by having three main meals and avoiding snacks. Participants were advised to aim for 2093–4186 kJ (500–1000 kcal) deficit compared to the Thais’ Dietary Reference Intake (7325.5 kJ [1750 kcal] and 8790.6 kJ [2100 kcal] for females and males, respectively) by using methods such as calorie counting, portion control, food exchange and reading food labels. This session also covered healthy eating tips to lose weight such as limiting intake of sugary food and beverages as well as fried and fatty foods; increasing intake of fruits and vegetables; substitution of red meat with white meat or fish; drinking a glass of water before meals and chewing food slowly while eating. In addition, participants were given advice about how to cope with situations or behaviours leading to excessive energy intake. Participants were also encouraged to inform their family members and friends of their intention to lose weight in order to create a supportive environment. All participants were advised to increase physical activity, aiming for a minimum 150 min of moderate-intensity exercise per week and/or 10,000 steps per day. Participants were also instructed to keep a food diary (2 weekdays and 1 weekend day) every week throughout the study period.

The subsequent four sessions at weeks 2, 4, 8 and 12 were a 30-min individual counselling with a dietitian. Participants were weighed and given individualised dietary and physical activity advice based on their reported food diaries and weight-loss achievement.

In addition to the lifestyle education, participants in the LEI + MR group were supplied with high-protein MR (Slimwell®, Benswell Corporation, Bangkok, Thailand) and instructed to replace two main meals daily with one sachet per meal (2 sachets per day), which were either breakfast, lunch or dinner throughout the 12-week period. For the remaining meal, they were advised to eat food that was low in fat and sugar. Each sachet of the MR powder contained 912.5 kJ (218 kcal), 46% of carbohydrate (24.95 g), 29% of protein (15.92 g) and 25% fat (6.06 g), and was dissolved in 250 mL of warm water. Sufficient MR were supplied at each study visit until the participants’ next scheduled visit. Compliance to the MR intake was assessed based on the numbers of returned empty sachets and also during the individual counselling with dietitian.

### Outcomes measurement

BW, WC, BP and pulse rate (PR) were recorded at every study visit throughout the 12-week period. BW was also measured at weeks 38 and 64. FPG, total cholesterol (TC), TG, HDL-c and low-density lipoprotein cholesterol (LDL-c) were measured at baseline, 4, 8 and 12 weeks. HbA_1c_, fasting plasma insulin, urine microalbumin and body composition were only measured at baseline and 12 weeks.

### Anthropometric measurement

BW was measured without shoes and heavy accessories while wearing indoor clothing using a calibrated weighing scale (TANITA® BC-418, Tanita corp., Tokyo, Japan) to the nearest 0.1 kg. Similarly, height was measured using a stadiometer (TANITA® WB-3000, Tanita corp., Tokyo, Japan) to the nearest 0.01 m. BMI was calculated as BW (kg) divided by height in metres squared. Percentage weight loss (%WL) was determined using the following formula: %WL = ([baseline BW − BW at each study visit]/baseline BW) × 100. WC was measured using a non-stretchable tape with measurement taken at a horizontal line midway between the highest point of iliac crest and the lowest ribs. Body composition were measured using BIA (TANITA® BC-418, Tanita corp., Tokyo, Japan).

### Metabolic and cardiovascular indices measurement

BP and PR were obtained using an electronic sphygmomanometre (Terumo Elemano [ES-H55], Medaval, New Jersey, United States) in a comfortable sitting position after at least 15-min rest. Blood sampling was undertaken following a 12-h overnight fast. TC, HDL-c, LDL-c, TG, glucose, insulin, urine microalbumin and urine creatinine were analysed with a biochemical auto-analyser (Cobas® 8000 Modular Analyser Series, Roche Diagnostics, Indianapolis, United States). HbA_1c_ was determined using Cobas Integra® 800 analyser, Roche Diagnostics, Indianapolis, United States. Homeostatic model assessment of insulin resistance (HOMA-IR) was calculated as: HOMA-IR = (FPG × fasting insulin)/22.5 in molar units.

### Statistical analysis

Sample size was determined by using BW reduction at 12 weeks as the primary outcome. A previous study^16^ showed 6.4 ± 6.9 kg weight loss in the intervention group and 3.1 ± 7.1 kg in the control group. In order to detect this difference with 80% power and 0.05 of type I error, 69 participants for each arm were required. Shapiro–Wilk test was used to test normality of all variables before performing statistical analysis. For continuous data, normally distributed data were reported as mean ± SE and non-normally distributed data were presented as median (25th, 75th percentiles). The categorical data were described as percentages, and the comparison between groups was performed by *χ*^2^ tests. Paired-sample and unpaired *t*-tests were used to compare normally distributed data within and between groups, respectively, and Mann–Whitney tests were used for non-normally distributed data.

Mixed models were used to compare the effect of treatment over time on quantitative outcomes using their baseline values as covariates. Model selection was based on the Bayesian Information Criterion. Assumptions for mixed models (e.g. normality of error terms) were checked thoroughly using the residual plots. Comparison of the predicted outcomes between two treatments at each time point was also performed. The analysis was conducted by a modified intention-to-treat (ITT) approach for participants who completed the study. For those completed the study with ≥80% of MR consumption, per-protocol (PP) analysis was conducted. Statistical analyses were performed by using SPSS for windows (version 24.0, Chicago, Illinois, USA) and SAS University edition. All tests were two-sided with a significance level at *p*-value < 0.05.

## Results

### Participant flow and baseline characteristics

**Fig. 1:**
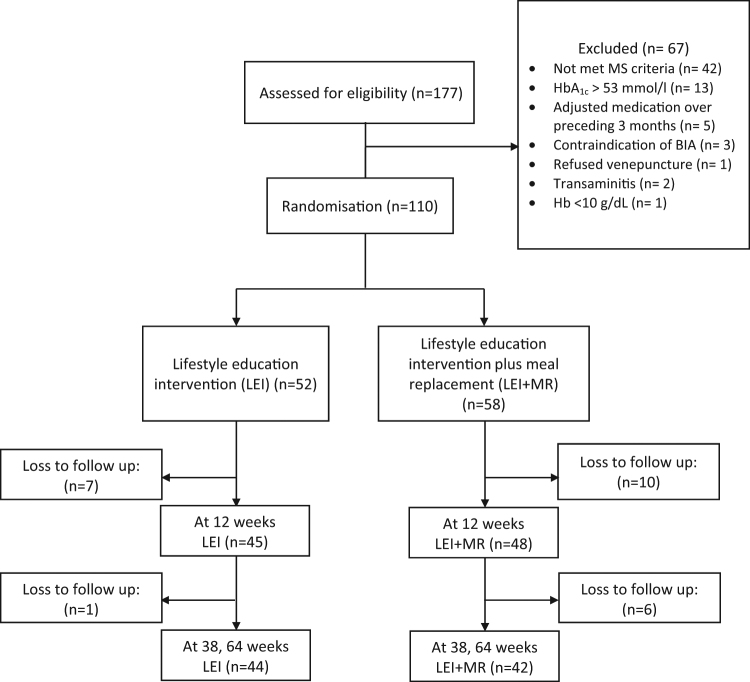
**Flow diagram of participant enrolment, consent, randomisation and associated timeline**

Figure 1 describes participant flow. A total of 177 subjects provided informed consent and underwent baseline assessments. Of these, 67 subjects were excluded with majority due to unmet MS criteria (62.7%), uncontrolled T2DM (19.4%) and had medication adjusted over preceding 3 months (7.5%). Overall, 52 subjects were assigned to LEI and 58 to LEI + MR. Overall, participants’ mean ± SE age was 42.5 ± 1.1 years old, BMI was 34.6 ± 0.6 kg/m^2^ and 83% were female. A total of 54.5% had impaired FPG, 47.3% hypertension, 33.6% dyslipidaemia and 10% were diabetics. There were no significant differences in the baseline characteristics between both groups (Table 1). At 12-week, 86.5 and 82.8% of LEI and LEI + MR completed the outcomes assessment. Whereas, 84.6% of LEI and 72.4% of LEI + MR had their BW measured at week 38 and 64, respectively (Fig. 1).

**Table 1:** Baseline characteristics

	LEI	LEI + MR	*p*-value
	(*n* = 52)	(*n* = 58)	
Age, years	43.2 ± 11.9^a^	41.8 ± 11.8^a^	0.53
Female, %	78.8	86.2	0.31
Body weight, kg	84.6 (75.5, 106.2)	84 (73.9, 97.7)	0.44
Body mass index, kg/m^2^	33.1 (30, 38.3)	32 (30.4, 37.6)	0.88
Waist circumference, cm	101.8 (97, 115.4)	103.5 (96.5, 114.4)	0.96
Fat mass, %	39.1 ± 0.9^a^	39.6 ± 0.9^a^	0.68
Co-morbidities			
Impaired fasting plasma glucose, %	48.1	60.3	0.20
Diabetes, %	7.7	12.1	0.45
Hypertension, %	51.9	43.1	0.36
Dyslipidaemia, %	38.5	29.3	0.31
Obstructive sleep apnoea, %	9.6	6.9	0.73
Non-alcoholic fatty liver disease, %	5.8	1.7	0.34

Numbers are median (P25, P75)

^a^Numbers are means ± SE

### Weight loss

**Fig. 2:**
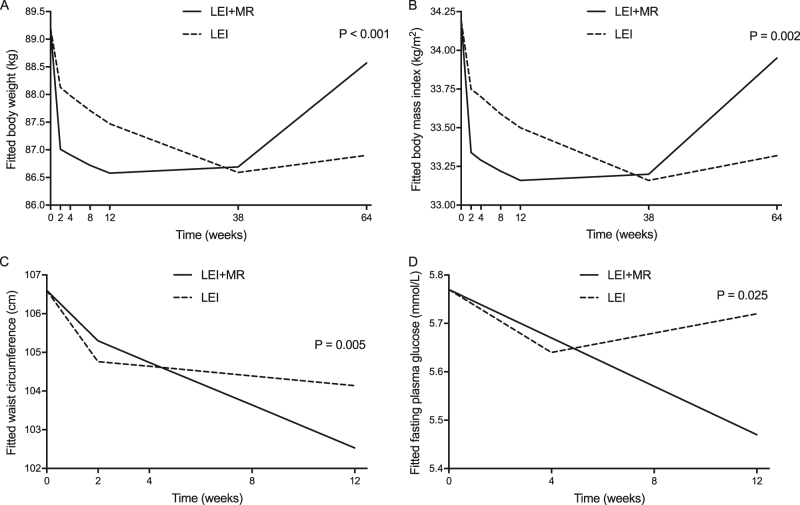
Mixed models comparing the outcomes between lifestyle education intervention (LEI) group and LEI with meal replacement (LEI + MR) group, **a** mean body weight (kg) in modified intention-to-treat analysis, **b** mean body mass index (kg/m^2^) in modified intention-to-treat analysis, **c** mean waist circumference (cm) in modified intention-to-treat analysis, **d** mean fasting plasma glucose (mmol/L) in per-protocol analysis

**Fig. 3:**
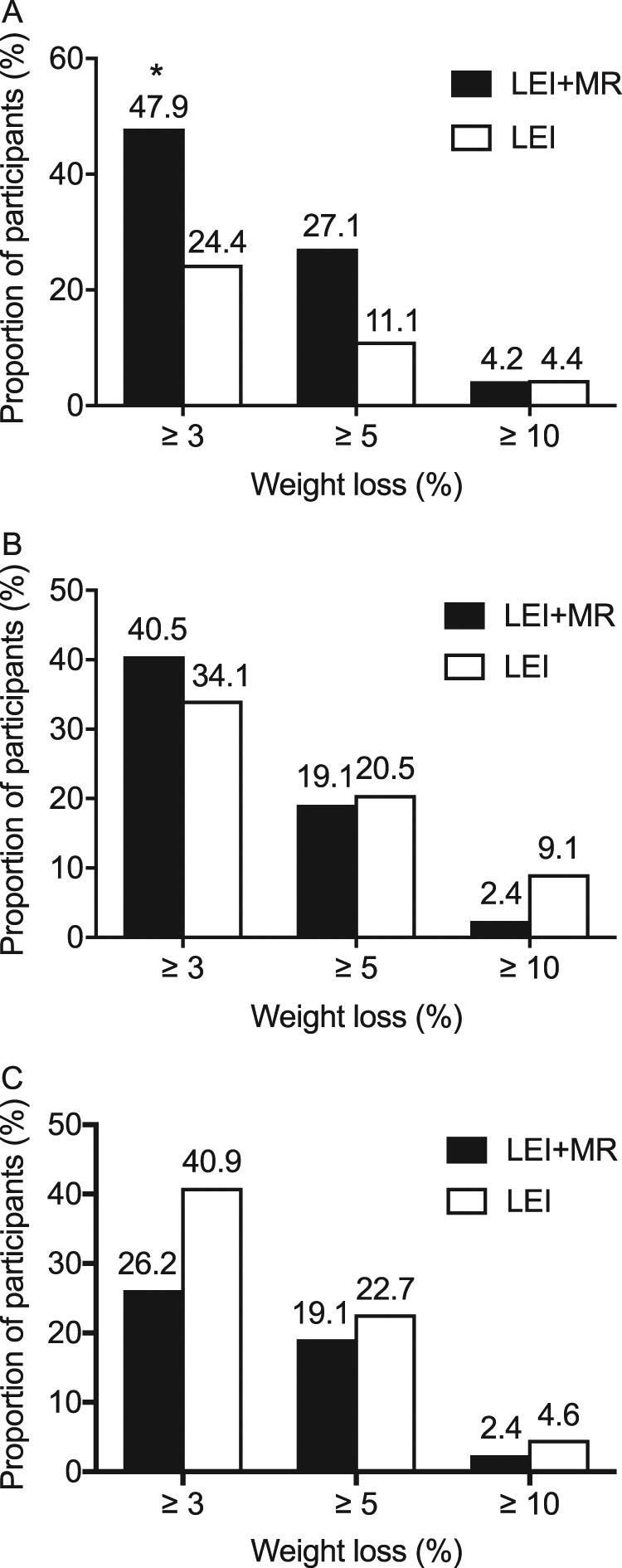
Proportion of individuals achieving ≥3, ≥5 and ≥10% weight loss, **a** at 12 weeks, **b** at 38 weeks and **c** at 64 weeks, *p = 0.019 between groups, LEI = lifestyle education intervention, LEI + MR = LEI with meal replacement

Both the LEI and the LEI + MR reduced weight and BMI significantly at the end of 12-week intervention compared to baseline (Table 2). Weight loss was observed in the majority of participants, 90% of LEI + MR group and 76% of LEI group. From the 2-week visit until the end of the 12-week intervention, the LEI + MR group exhibited greater weight loss than the LEI group (Fig. 2a, b). At 12 weeks, LEI + MR achieved greater %WL than LEI (*p* < 0.05, Table 2). Examination of the percentage of participants within each group achieving ≥3, ≥5, ≥10% at the end of the intervention period are shown in Fig. 3a. For each %WL category, the percentage of participants was greater in the LEI + MR group however this only reached significance in the ≥3% category (*p* < 0.05).

**Table 2:** Outcomes at 12 weeks by modified intention to treat analysis

	LEI (*n* = 45)				LEI + MR (*n* = 48)				*p*-value between groups
	Baseline	12 weeks	Changes	*p*-value	Baseline	12 weeks	Changes	*p*-value	
Anthropometric changes									
Body weight, kg	84.4 (75.6, 105)	81.5 (74.6, 104.8)	−1.4 (−2.75, 0)	0.01	82.8 (72.9, 94.1)	80.3 (69.8, 90.6)	−2.3 (−4.93, −0.68)	<0.01	0.01
Body mass index, kg/m^2^	33.1 (29.8, 38.3)	32.6 (28.9, 37.7)	−0.49 (−1.0, −0.00)	<0.01	31.9 (30.4, 36.1)	30.6 (28.6, 35.4)	−0.94 (−1.77, −0.3)	<0.01	0.01
Weight loss, %			1.53 (−0.01, 3.05)				2.86 (1.07, 5.7)		0.01
Waist circumference, cm	101 (97, 115.3)	99 (94.3, 115.3)	−2.5 (−5.5, 0.25)	<0.01	103.3 (96.6, 111.8)	99 (92.3, 108.5)	−3.25 (−6.0, −1.13)	<0.01	0.13
Fat mass, %	38.6 ± 1^a^	37 ± 1.0^a^	−0.8 (−2.2, 0.05)	<0.01	38.9 ± 1^a^	37.8 ± 0.9^a^	−1 (−1.7, −0.03)	<0.01	0.83
Fat mass, kg	32 (26.1, 42.7)	30.9 (23.4, 40.9)	−1.05 (−2.62, −0.19)	<0.01	29.8 (26.7, 39.9)	28.2 (24.9, 38.7)	−1.58 (−3.52, −0.57)	<0.01	0.20
Fat-free mass, kg	52.5 (46.2, 60.5)	51.7 (46.2, 60.1)	0.23 (−1.16, 1.31)	0.46	49.1 (45.6, 56.9)	48.6 (44.5, 56.6)	−1.01 (−1.85, 0.61)	0.02	0.02
Fat mass index, kg/m^2^	13 (10.4, 15.2)	12.6 (9.5, 14.1)	−0.44 (−0.98, −0.07)	<0.01	11.9 (10.9, 14.9)	11.5 (9.8, 14.3)	−0.59 (−1.29, −0.24)	<0.01	0.21
Fat-free mass index, kg/m^2^	20.4 (18.5, 22.9)	20.7 (18.4, 22.8)	0.09 (−0.46, 0.5)	0.47	19.9 (18.5, 21.8)	19.7 (18.3, 21.1)	−0.39 (−0.77, 0.24)	0.02	0.02
Metabolic and cardiovascular indices									
Systolic blood pressure, mmHg	129 (117, 141)	124.9 ± 1.9^a^	−5.11 ± 2.06^a^	0.02	129 (114, 141)	122.5 ± 2.3^a^	−5 ± 2.22^a^	0.03	0.97
Diastolic blood pressure, mmHg	75 (71, 84)	73.8 ± 1.7^a^	−4.53 ± 1.6^a^	0.01	77 (68, 85)	71.1 ± 1.5^a^	−5.65 ± 1.59^a^	<0.01	0.62
Pulse rate, bpm	77 (72, 85)	75 (70, 84)	−3.0 (−9.0, 5.5)	0.49	77 (72, 85)	75 (68, 82)	−1.5 (−10, 4)	0.03	0.68
Fasting plasma glucose, mmol/L	5.55 (5.16, 6.11)	5.61 (5.08, 5.97)	0.00 (−0.25, 0.19)	0.54	5.77 (5.34, 6.22)	5.44 (5.11, 5.81)	−0.22 (−0.56, 0.06)	<0.01	0.03
Fasting insulin, pmol/L	131.6 (76.1, 217.9)	146.6 (100, 212.2)	18.1 (−27.4, 51.8)	0.10	123.6 (70.5, 181.2)	97.9 (70.7, 160.3)	−14.2 (−51.2, 30.8)	0.22	0.03
HOMA-IR	4.49 (2.41, 7.83)	4.59 (3.47, 7.3)	0.55 (−0.83, 1.76)	0.14	4.7 (2.5, 6.67)	3.27 (2.42, 6)	−0.7 (−2.41, 1.09)	0.22	0.02
Haemoglobin A1c, mmol/mol	40.1 ± 0.7^a^	41 (37.7, 44.3)	1.09 (−0.55, 2.19)	0.03	41.2 ± 0.7^a^	39.9 (37.7, 43.2)	0.00 (−2.19, 1.09)	0.30	0.01
Total cholesterol, mmol/L	5.04 ± 0.15^a^	4.99 ± 0.13^a^	−0.13 (−0.36, 0.19)	0.55	5.18 ± 0.14^a^	4.99 ± 0.13^a^	−0.2 (−0.52, 0.21)	0.05	0.31
Triglyceride, mmol/L	1.53 (1.15, 2.1)	1.39 (1.15, 1.92)	0.00 (−0.38, 0.26)	0.21	1.7 (1.29, 2.15)	1.55 (1.1, 2.22)	−0.12 (−0.27, 0.08)	0.45	0.46
HDL-c, mmol/L	1.14 (1.01, 1.27)	1.11 (0.96, 1.3)	−0.03 (−0.12, 0.08)	0.35	1.18 (1.04, 1.45)	1.15 (1.02, 1.37)	−0.01 (−0.13, 0.08)	0.96	0.96
LDL-c, mmol/L	3.09 ± 0.13^a^	3.12 ± 0.11^a^	0.08 (−0.27, 0.35)	0.70	3.13 ± 0.15^a^	2.96 ± 0.13^a^	−0.23 (−0.43, 0.21)	0.08	0.07
Microalbuminuria, mg/g Cr	7.9 (4.55, 24.2)	5.8 (3.4, 23.4)	−1.1 (−6.55, 1.85)	0.34	10.35 (4.18, 21.08)	8.05 (4.75, 15.73)	−0.55 (−7.23, 2.78)	0.69	0.59

Numbers are median (P25, P75)

*Fat mass index* computed by Fat mass (kg)/ height (m)^2^, *Fat-free mass index* computed by fat-free mass (kg)/ height (m)^2^, *HOMA-IR* homeostatic model assessment of insulin resistance, *HDL-c* high-density lipoprotein cholesterol, *LDL-c* low-density lipoprotein cholesterol, *Cr* creatinine

^a^Numbers are means ± SE

At the 38 weeks, 26 weeks after the end of the study period, the mean BW was significantly less than baseline in both groups (−1.8 [−4.13, 0.43] kg [*p* < 0.01] vs. −0.8 [−4, 1.05] kg [*p* < 0.05], LEI + MR vs. LEI, respectively), but no significant difference between groups was observed (Fig. 2a). The percentage of participants in each %WL category is shown in Fig. 3b. Examination of BW at 64 weeks, 52 weeks following the end of the intervention, revealed no overall difference in BW compared to baseline and between groups (−1.05 [−3.93, 1.8] kg vs. −0.4 [−3.23, 1.7] kg, LEI vs. LEI + MR, respectively) (Fig. 2a). The percentage of participants in each %WL category is shown in Fig. 3c. However, despite the lack of an effect at group levels, there was an individual variable effect as shown in Fig. 3.

### Waist circumference

At the end of the 12-week intervention period, WC was significantly decreased compared to baseline in both groups but with a greater reduction over time observed in the LEI + MR group compared to the LEI group (Table 2, Fig. 2c; *P* < 0.01). There was a significant correlation between weight loss and the decrease in WC (r = 0.457, *P* < 0.001).

### Body composition

Participants in both groups experienced a significant reduction in FM from baseline with no significant difference between groups. However, there was a significant reduction in fat-free mass (FFM) in the LEI + MR group compared to baseline (*p* < 0.05), but not in LEI (Table 2).

### Glycaemic control

FPG was significantly reduced in LEI + MR at 12 weeks compared to baseline but not in LEI group, consequently LEI + MR group experienced a significantly lower level of FPG than LEI, *p* < 0.05 (Table 2). The difference between groups over time was significant in PP analysis (Fig. 2d), but not in modified ITT analysis. Overall, 43% of subjects with impaired FPG at baseline in LEI + MR returned to normal FPG compared to only 19% of the LEI. No subjects in the LEI + MR changed from normal FPG to impaired FPG, but 14% of subjects in the LEI developed impaired FPG (Table 3). A significant correlation between weight loss and the decrease in FPG was observed (*r* = 0.252, *p* < 0.05). HbA_1c_ significantly increased in LEI (*p* < 0.05) compared to baseline but not in LEI + MR, making the level significantly lower in LEI + MR than LEI at 12 weeks. In addition, there was a significant correlation between weight loss and the decrease in HbA_1c_ level (*r* = 0.448, *p* < 0.001). The levels of fasting insulin and HOMA-IR were significantly lower in LEI + MR than LEI at the end of the 12-week intervention period, *p* < 0.05 (Table 2).

**Table 3:** Glycaemic status at 12 weeks

	LEI	LEI + MR	*p*-value
	(*n* = 45)	(*n* = 48)	
Glycaemic status			
IFG returned to normal	4 (19%)	12 (42.9%)	0.04
Normal turned to IFG	3 (14.3%)	0 (0%)	0.07
Remained normal	18 (85.7%)	13 (100%)	0.21
Remained IFG	17 (81%)	16 (57.1%)	0.71

*IFG* impaired fasting plasma glucose

### Blood pressure, lipids and microalbuminuria

Systolic and diastolic BP were both significantly reduced in both groups with no differences between groups (Table 2). There were no significant changes in TG, HDL-c, LDL-c and microalbuminuria (MAU) between baseline and at 12 weeks in either group or between groups (Table 2). PR and TC level in LEI + MR at 12 weeks were significantly lower than baseline (Table 2).

### Changes in the incidence of MS

At the end of the 12-week intervention period, 13.3% of participants in LEI (*n* = 6/45) and 18.8% (*n* = 9/48) in LEI + MR no longer fulfilled MS criteria. The difference was not significant between groups.

### Compliance and adverse effects of MR

Seventy-four percent of participants in LEI + MR group (*n* = 43/58) ingested ≥80% of the total amount of MR, suggesting that MR was tell tolerated, and there was no report of any adverse events in either group.

### PP analysis

All results were consistent with the modified ITT analysis except the FPG.

## Discussion

Our study demonstrates that an LEI alone in Thai adults with obesity and MS leads to weight loss and improvement in MS. Moreover, that LEI combined with partial MR leads to greater weight reduction and improvement in glycaemic indices. To the best of our knowledge, this is the first trial of its kind undertaken in a Thai population.

Our findings of modest weight loss and benefits in MS in both groups compared to baseline, show that a pragmatic LEI is an effective weight-loss strategy for Thai patients with obesity, in agreement with the previous study undertaken by Karintrakul et al.^14^

BW reduction was significantly greater in LEI + MR than LEI alone. This difference could be due to a lower total energy intake in the LEI + MR compared to the LEI group, as a consequence of replacing two of the daily main meals with MR. Each MR contained 912.5 kJ (218 kcal) compared to the 2093 kJ (500 kcal) that is usually consumed in a typical Thai meal^28^. Furthermore, the MR used in our study possesses high protein as the high dietary protein is thought to enhance satiety through various mechanisms^29–31^.

Consistent with other studies^11,12^, after cessation of the intervention, subjects in both groups regained weight at 64 weeks. This finding emphasises the need for ongoing support. Indeed, the recent DiRECT study, which including monthly individually tailored calorie prescription and support weight stabilisation, reported only 1.9 kg of weight regain during the weight maintenance phase up to 52 weeks^32^. In addition, one MR per day could potentially have a role in weight maintenance, this was an option for participants in DiRECT. Even though the difference in BW between groups at 64 weeks is not statistically significant, it appears that LEI + MR tends to regain weight more than LEI. This could be due to a greater reliance on MR rather than lifestyle modification in the LEI + MR group. On the other hand, the only intervention offered to the LEI group was LEI. Hence, after cessation of the intervention, subjects in LEI perhaps may have continued with their lifestyle modification to a greater extent than LEI + MR. In addition, the more extreme outliers in LEI who lost substantial weight may potentially deviate the mean of the weight reduction observed at 64 weeks.

WC is a strong predictor of T2DM, CVD risk factors and CVD events^33^. A previous study has shown that every centimetre increase in WC was associated with a 2% increase in CVD risk^34^. Similarly, a study undertaken in Thailand has also demonstrated that normal weight individuals with central obesity were 2.03 times more likely to have at least one CVD risk factor compared to normal weight individuals without central obesity^35^. In addition, evidence has shown that a reduction in WC resulted in a decline in CVD risk^36^. In our study, there was a significant decrease in WC in both groups during the intervention but the LEI + MR group exhibited a significantly greater reduction. This finding implies that patients could benefit from the reduction in CVD risk.

Glycaemic control (FPG and HbA_1c_) and insulin sensitivity (fasting insulin level and HOMA-IR) improved significantly more in LEI + MR than LEI at 12 weeks. The percentage of subjects with reversal of impaired FPG at 12 weeks was also significantly greater in LEI + MR than LEI. This could be explained by the greater weight loss, lower energy and CHO consumption in LEI + MR compared to LEI and the high-protein MR per se. A study undertaken by Daniel König et al. demonstrated that ingestion of high-protein/low glycaemic index MR promoted greater fat oxidation, and thus improved insulin sensitivity compared to a high glycaemic index/low protein diet^29^. The normalisation of FPG seen in our study is likely to prevent the future development of diabetes, as observed in the Diabetes Prevention Program Outcomes Study (DPPOS) demonstrating that the regression from IPG to normal glucose regulation reduced long-term diabetes increase by 56%^37^.

Elevated BP is common in patients with obesity and MS. Our findings support the effectiveness of lifestyle modification in reducing BP because both groups exhibited significant reduction in systolic and diastolic BP. The improvement in lipid profiles in our study was not significant. This could be due to the normal or only slightly high levels of the lipid profiles at baseline. Moreover, patients in the present study only achieved a modest weight loss, whereas at least 5–10% or 3–5 kg of weight reduction is required to achieve therapeutic targets^9,33^.

Subjects in both groups lost FM at 12 weeks compared to baseline, but no significant difference between groups. It is known that both FM and FFM loss occur during the weight-loss period^38^. We found that at 12 weeks FFM in the LEI + MR group was significantly lower than in the LEI group. This finding may at least be explained by the greater weight loss achieved in LEI + MR than LEI. Alternatively, those subjects in LEI might have been more physically active than LEI + MR, although we did not monitor subjects’ compliance towards the recommended physical activity.

With regard to the applicability of MR, the use of MR was well-tolerated as no adverse effects were reported. Nonetheless, the cost is a major disadvantage. Given that the average income per month of individuals in Thailand was 13,878 THB^39^ in 2017 and the cost of MR was 11,800 THB monthly, MR could only be an option for people who are able to afford the cost.

There are several strengths of the present study. First, the LEI used was pragmatic and less expensive than intensive lifestyle interventions, a best practice in the Western world. Hence, it is probably more feasible for low- and middle-income countries where resources and manpower are limited. Second, allowing subjects to decide which meals were suitable for them to take MR was also another strength as it is flexible and feasible on a day-to-day basis. Third, we demonstrated that after cessation of the interventions, there was a weight regain in long-term follow-up until 64 weeks. In addition, the current guidelines for management of overweight and obesity in adults recommended at least 3–5 %WL in 2013 AHA/ACC/TOS guideline^9^ and 5–15 %WL in 2016 AACE/ACE guideline^33^ to achieve metabolic benefit and reduce the risk of developing T2DM. Nevertheless, despite only a modest weight reduction achieved in the present study (1.5–2.9%), significant advantages were observed regarding MS components, glycaemic control and insulin sensitivity. Importantly, this modest weight loss resulted in reduction in the number of participants fulfilling MS criteria by 16% and led to the regression of IFG.

Several limitations are worth noting. First, the accuracy of the food record is limited since it tends to underestimate the amount of actual intake. We thus used it primarily for self-feedback to subjects as a part of behavioural modification. Second, participants’ compliance towards the recommended physical activity levels was not assessed. Nevertheless, during the individual follow-up session, the dietitian reviewed the food diary and interviewed subjects about the actual intake and physical activity to lessen these limitations. Finally, owing to the limited funding and unexpected excessive number of excluded subjects during the baseline assessment, there were only 110 subjects in both groups after randomisation, not attaining the sample size determination. Nonetheless, the difference of weight reduction between groups achieved statistical significance.

Future studies should examine a total MR regimen, similar to the DiRECT study, and the advantages and disadvantages of the MR in the longer term, particularly with respect to weight-loss maintenance. Furthermore, the mechanisms behind the greater improvement of weight reduction, glycaemic control, insulin resistance and MS in the LEI + MR group need to be elucidated.

In conclusion, LEI and LEI + MR are acceptable and practicable for Thai patients with obesity and MS and lead to improvement in MS components and glycaemic indices and modest weight reduction. The LEI + MR group exhibited greater benefits at 12 weeks. Future longer term studies with ongoing support is now warranted.
